# The contribution of pain and disability on the transition from acute to chronic pain-related TMD: A 3-month prospective cohort study

**DOI:** 10.3389/fpain.2022.956117

**Published:** 2022-08-26

**Authors:** Ana Miriam Velly, Sherif M. Elsaraj, Jack Botros, Firoozeh Samim, Zovinar der Khatchadourian, Mervyn Gornitsky

**Affiliations:** ^1^Department of Dentistry, Jewish General Hospital, Montreal, QC, Canada; ^2^Faculty of Medicine and Oral Health, McGill University, Montreal, QC, Canada; ^3^Lady Davis Institute for Medical Research, Montreal, QC, Canada; ^4^Alan Edwards Pain Management Unit, Montreal General Hospital, Montreal, QC, Canada; ^5^Department of Dentistry, Montreal General Hospital, Montreal, QC, Canada

**Keywords:** risk factors, temporomandibular disorders (TMD), acute to chronic TMD pain transition, pain intensity, risk difference

## Abstract

Although most cases of pain-related temporomandibular disorders (TMD) are mild and self-limiting, about 10% of TMD patients develop severe disorders associated with chronic pain and disability. It has been suggested that pain intensity contributes to the transition from acute to chronic pain-related TMD. Therefore, the aims of this current prospective cohort study were to assess if pain intensity, pain always being present, pain or stiffness on awakening, jaw activities, and interference, were associated with the transition from acute to chronic pain-related TMD at 3 months of follow-up. One hundred and nine participants, recruited from four clinics in Montreal and Ottawa, received examinations and completed the required instruments at baseline and at the 3rd month of follow-up. In a multivariable analysis including sex, age, characteristic pain index (CPI) (OR = 1.03, 95%CI = 1.01–1.06, *P* = 0.005), moderate to severe average pain intensity (OR = 3.51, 95%CI = 1.24–9.93, *P* = 0.02), disability points score (OR = 1.29, 95%CI = 1.06–1.57, *P* = 0.01), interferences (ORs = 1.30–1.32, *P* = 0.003–0.005), screening score (OR = 1.37, 95%CI = 1.08–1.76, *P* = 0.01), and pain always present (OR = 2.55, 95%CI = 1.08–6.00, *P* = 0.03) assessed at first-visit were related to the transition outcome at the 3rd month of follow-up. Further, we found that if 4 patients with acute pain-related TMD on average were exposed to these risk factors at baseline, 1 would have the transition from acute to chronic pain at 3 months of follow-up. Results indicate that these factors are associated with the transition from acute to chronic pain-related TMD, and therefore should be considered as important factors when evaluating and developing treatment plans for patients with pain-related TMD.

## Introduction

Temporomandibular disorders (TMD) are prevalent among the general population with a percentage ranging between 5 and 12% (1). More than one-third of these patients suffer from high levels of pain, and more than 10% are highly disabled (2–4). Despite the various treatment modalities used for these conditions (5), more than 1 subject out of 3 with the first onset of TMD continues to have persistent pain at a 6-month follow-up (6–8).

Pain intensity was found to increase the risk of TMD (9) as well as its persistence (10–12). TMD patients with higher Characteristic Pain Index (CPI) scores have a higher risk of transition from acute to chronic pain at 6 months of follow-up (13). A borderline association was found with the Graded Chronic Pain Scale (GCPS) III–IV. However, as the International Association for the Study of Pain (IASP) updated the definition of chronic pain as “pain that lasts or recurs for longer than 3 months” (14), it is not clear whether pain intensity contributes to the transition from acute to chronic pain-related TMD or with its persistence (15). The National Institute of Health (NIH) reported “we do not fully understand how acute progresses to chronic pain at any level, from molecular to behavioral”^1^.

In 2015, the Acute to Chronic TMD Transition (ACTION) program was initiated by Dr. Ana Velly and her team. The overall aims of this program are to determine the risk factors that contribute to the transition from acute to chronic pain-related TMD and its persistence. Therefore, regarding the transition from acute to chronic pain-related TMD, the aims of this current cohort study were to assess if pain intensity, pain always present, pain or stiffness in the jaw on awakening, jaw activities, and interference, assessed at first-visit were associated with the transition from acute to chronic pain-related TMD at 3 months of follow-up. Our hypotheses were that patients exposed to more severe pain intensity, persistent pain, disability, and with a greater screening TMD score have greater transition risk. To our knowledge, no previous studies investigated the risk factors associated with the transition from acute to chronic pain at 3-month follow-up.

## Methods

### Study design

This 3-month prospective cohort study is a part of the ACTION project assessing the risk factors for the transition and the persistence of pain-related TMD. This project was approved by the McGill Institutional Review Board in Montreal, Quebec (approval number: A12-M113-14A) and by the Dental Specialists Group in Ottawa, Ontario (approval number: 240-400) and follows the Helsinki declaration. All participants agreed to participate in this study and signed the consent form. This article follows the strengthening the reporting of Observational Studies in Epidemiology guidelines for methodology and statistical analyses (16).

### Study population eligibility and enrollment

Patients were eligible for this study if they received the diagnoses of pain-related TMD (muscle and/or joint pain) according to the Diagnostic Criteria (DC) or Research Diagnostic Criteria (RDC) of TMD (17–19), presented pain-related TMD for 3 months or less, and were aged between 18 and 85. The IASP new definition of chronic pain was followed to define acute pain-related TMD (14). RDC and DC protocols have high validity and reliability and are considered the gold standard for the diagnosis of pain-related TMD (17, 19).

The examination was undertaken by a dentist experienced in orofacial pain at each site. Patients completed a series of questions assessing the pain-related TMD symptoms following the RDC and DC protocols (17, 19); Graded Chronic Pain Scale (GCPS pain grades I, II, III, IV) (2).

Patients were excluded if they: (i) had cancer; (ii) did not understand English or French; (iii) had no access to a telephone; or (iv) could not provide informed consent.

Participants were enrolled at four sites: (i) the Jewish General Hospital general dental clinic; (ii) the Faculty of Dentistry of McGill University oral diagnosis clinic; (iii) Montreal General Hospital?; and (iv) the Dental Specialists ?Group TMD-specialized clinic. Recruitment started in 2015 and ended in December 2021.

### Study outcome

The transition outcome was defined as an indicator that the pain duration had crossed over 3 months. The pain outcome—Characteristic pain intensity (CPI)—was assessed through the GCPS (2) measurement on follow-up examinations or calls to patients, 1–2 weeks after their pain duration reaches 3 months. CPI is the average of 0–10 ratings of current, worst, and average pain in the last 30 days multiplied by 10 (20).

### Assessment of potential risk factors, confounders, and effect modifiers

The potential risk factors were CPI, current, worst, average pain intensity, days with usual activity limitation, pain interference on activities: daily, recreational, social, and familial; and interference on ability to work, all assessed with GCPS (2, 20). Moreover, items from the screener instrument were also considered as potential risk factors. The potential confounders and effect modifiers were age, sex, and pain duration. All potential risk factors, confounders, and effect modifiers were assessed on the first-visit (baseline).

### Statistical analysis

Descriptive and bivariate analyses were used to describe the characteristics of the acute cohort. To determine the risk factors for the transition from acute to chronic pain at the 3rd month follow-up, we performed a series of crude and multivariable logistic regression analyses (PROC logistic, SAS) adjusted by sex, age, and pain duration (number of months) at baseline. Odds ratio (OR) and their 95% confidence intervals (CI) were estimated in these analyses. Further, since the incidence of the transition was >10%, the following formula was used to approximate the risk ratio (RR) from the adjusted OR: RR = OR/[(1 – *P*_0_) + (*P*_0_ × OR)], where *P*_0_ indicates the incidence of the transition outcome in the non-exposed group (21). We estimated the area under the receiver operating characteristic curve (AUROC) for each predictor.

Further, the risk difference was calculated as the variation of the transition risk in the exposed population and the non-exposed group. The crude attributable fraction was calculated as the risk difference divided by the exposed risk. Adjusted attributable fraction was calculated using this formula = pd^*^(RR_adjusted_ – 1)/RR_adjusted_, where pd is the proportion of patients with the transition and exposed to the risk factor and RR is the relative risk of transition associated with the risk factor (22).

The number needed to harm was calculated as the inverse of the attributed fraction. All analyses were performed using the statistical software package SAS (version 9.4), with the significance level for type I error set at the 0.05 level.

## Results

**Figure 1:**
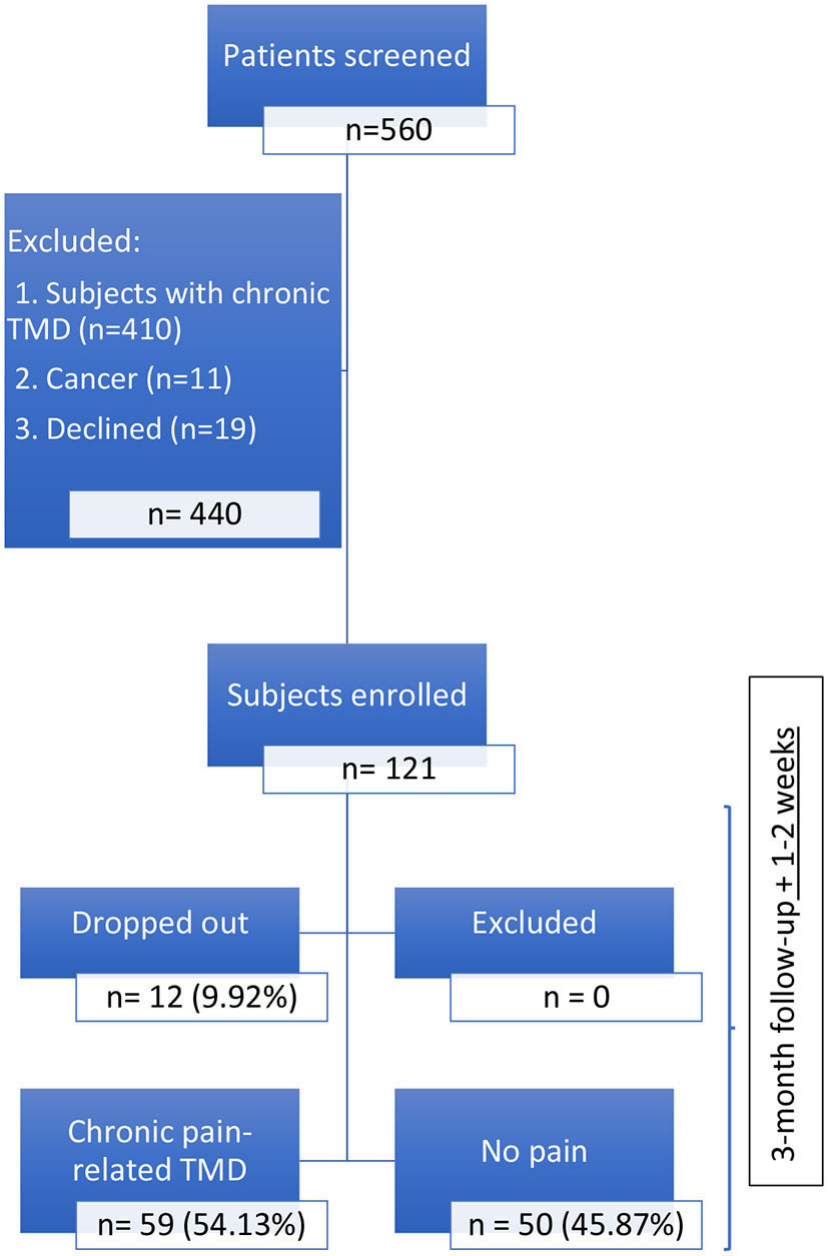
Participant flow diagram.

At baseline, 121 patients were included in the acute pain-related TMD cohort: 71 (58.68% from the Jewish General Hospital (JGH) General dental clinic; 10 (8.26%) from the Faculty of Dentistry of McGill University oral diagnosis clinic; 7 (5.79%) from the Montreal General Hospital (MGH), and 33 (27.27%) from the Dental Specialists Group TMD-specialized clinic. One-hundred and nine (90.08%) patients completed the follow-up; and 12 (9.92%) dropped out: 10 from the JGH, and 2 from the Faculty of Dentistry of McGill University Oral Diagnosis clinic. Of those 109, 59 (54.13%) presented chronic pain-related TMD at the 3rd month follow-up (Figure 1); 44 (74.58%) females and 15 (25.42%) males, two patients did not complete the CPI at the first-visit.

Patients with higher levels of CPI assessed at the first visit presented higher odds for the transition from acute to chronic pain-related TMD at 3 months of follow-up than those with lower CPI, regardless of their age, sex, and pain duration—all unrelated to the transition risk (Table 1). CPI was associated with transition risk (RR = 1.02, 95%CI = 1.01–1.02, *P* < 0.05). Furthermore, both the crude and the adjusted analyses revealed that patients with moderate to severe CPI (≥45) at the first visit had a higher likelihood (OR_crude_ = 2.56, 95%CI = 1.11–5.90, *P* = 0.03; OR_adjusted_ = 2.88, 95%CI = 1.21–6.89, *P* = 0.02) and risk (RR_adjusted_ = 1.68, 95%CI = 1.12–2.13, *P* < 0.05) for acute to chronic transition at 3 months of follow-up than those with lower CPI, regardless of their sex (OR =2.13, 95%CI = 0.89–5.07, *P* = 0.09), age (OR = 1.01, 95%CI = 0.99–1.04, *P* = 0.27), and duration of the pain complaint (OR = 0.68, 95%CI = 0.42–1.09, *P* = 0.11). Specifically, this increased risk was related to average pain in the last 30 days (Table 2, RR = 2.21, 95%CI = 1.34–2.77, *P* < 0.05).

**Table 1:** Binary logistic regression analyses assessing the association between CPI, age, and sex and the acute to chronic transition at 3 months of follow-up.

**Potential risk factor**	**Category**	**Transition/ no transition**	**Crude model**		Multivariable model*	
			**OR (95% CI)**	***P*-value**	**OR (95% CI)**	***P*-value**
Characteristic pain intensity	Mean (SD)	61.78 (18.73)/ 51.02 (20.82)	1.03 (1.01–1.05)	0.008	1.03 (1.01–1.06)	0.005
Age	Mean (SD)	43.85 (17.35)/ 40.68 (16.23)/	1.01 (0.99–1.04)	0.33	1.02 (0.99–1.04)	0.18
Sex	Male, n (%)	15 (44.12)/ 19 (55.88)	1 (reference)	0.16	1 (reference	0.07
	Female, n (%)	44 (58.67)/ 31 (41.33)	1.80 (0.79–4.08)		2.32 (0.95–5.70)	
Pain duration (months)	Mean (SD)/ Min-Max	1.80 (0.88)	1 (reference)	0.29	1 (reference)	0.08
		1.62 (0.87)	0.80 (0.51–1.22)		0.65 (0.40–1.06)	

^*^Multivariable model includes characteristic pain intensity, age, sex and pain duration variables.

**Table 2:** Binary logistic regression analyses assessing the association between current, worst and average pain intensity and the acute to chronic transition at 3 months of follow-up.

**Potential risk factor**	**Category**	**Transition/** **no transition, *n* (%)**	**Crude model**		Multivariable model*		Multivariable model**	
			**OR (95% CI)**	***P*-value**	**OR (95% CI)**	***P*-value**	**OR (95% CI)**	***P*-value**
Current pain intensity	<4.5	27 (51.92)/ 25 (48.08)	1 (reference)	0.66	1 (reference)	0.13	*Not included*	
	≥4.5	32 (56.14)/ 25 (43.86)	1.19 (0.56–2.52)		0.46 (0.17–1.27)		
Worst pain intensity	<4.5	4 (21.05)/ 15 (78.95)	1 (reference)	0.003	1 (reference)	0.08	1 (reference)	0.09
	≥4.5	55 (61.11)/ 35 (38.89)	5.89 (1.81–19.21)		3.59 (0.85–15.08)		3.44 (0.82–14.44)	
Average pain intensity	<4.5	12 (31.58)/ 26 (68.42)/	1 (reference)	0.0008	1 (reference)	0.006	1 (reference)	0.02
	≥4.5	47 (66.20/ 24 (33.80)	4.24 (1.82–9.85)		5.17 (1.59–16.84)		3.51 (1.24–9.93)	

^*^Multivariable model includes current, worst, average pain intensity, sex (OR = 2.80, 95%CI = 1.07–7.28, P = 0.04); age (OR = 1.02, 95%CI = 0.99–1.05, P = 0.12); and pain duration (OR = 0.61, 95%CI = 0.37–1.03, P = 0.06).

^**^Multivariable model includes worst, average pain intensity, sex (OR = 2.77, 95%CI = 1.08–7.11, P = 0.04); and pain duration (OR = 0.61, 95%CI 0.37–1.02, P = 0.06).

The strength of the OR (Table 2), contrary to the RR (2.32, 95%CI = 0.88–3.81, *P* > 0.05) of worst pain was moderate; but both estimates were not statistically significant. The significant change in the magnitude of the worst pain OR was due to the correlation between average and worst pain intensity (*r* = 0.58, *P* < 0.0001). In addition, sex was associated with the transition odds (Table 2), but it did not modify the contribution of the average pain to the transition odds (β = −1.54, *P* = 0.24). Further, the results suggest that a large percentage of the transition was attributable to moderate to severe worst (35%) and average pain intensity (36%). The results also suggest that, on average, 3 patients exposed to either *moderate to severe* worst or average pain intensity at baseline would have the transition from acute to chronic pain at 3 months of follow-up (Table 3).

**Table 3:** Risk difference, attributed fraction and number need to harm associated with the transition from acute to chronic pain-related TMD at 3 months of follow-up.

**Potential risk factor assessed at first-visit**	**Category**	**Transition/** **no transition, n (%)**	**Risk (95% CI)**	**Risk difference (95% CI)**	**Crude attributed fraction**	**Adjusted attributed fraction**	**Number needed to harm based on CAF/AAF**
Characteristic Pain Intensity	<45	14 (40.0)/ 21 (60.0)	0.40 (0.24–0.56)	0.21 (0.02, 0.41)*	0.34	0.25	3/0.4
	≥45	44 (61.11)/ 28 (38.89)	0.61 (0.50–0.73)				
Current pain intensity	<4.5	27 (51.92/ 25 (48.08)	0.52 (0.38–0.66)	0.04 (−0.23, 0.15)	0.07	0.03	14/33
	≥4.5	32 (56.14)/ 25 (43.86)	0.56 (0.43–0.69)				
Worst pain intensity	<4.5	4 (21.05)/ 15 (78.95)	0.21 (0.03–0.39)	0.40 (0.19, 0.61)*	0.66	0.35	1.5/2.8
	≥4.5	55 (61.11)/ 35 (38.89)	0.61 (0.51–0.71)				
Average pain intensity	<4.5	12 (31.58)/ 26 (68.42)/	0.32 (0.17–0.46)	0.54 (0.45, 0.63)*	0.52	0.36	1.9/2.7
	≥4.5	47 (66.20)/ 24 (33.80)	0.66 (0.55–0.77)				
TMD screener	<3	10 (41.67)/ 14 (58.33)	0.42 (0.22–0.61)	0.16 (−0.06, 0.38)	0.28	0.16	3.6/6.3
	≥3	49 (57.65)/ 36 (42.35)	0.58 (0.47–0.68)				
Pain always present	Comes and goes	23 (41.07)/ 33 (58.93)	0.41 (0.28–0.54)	0.24 (0.06, 0.43)*	0.37	0.23	2.7/4.2
	Is always present	32 (65.31)/ 17 (34.69)	0.65 (0.52–0.79)				
Pain or stiffness on the jaw on awakening	No	11 (34.38)/ 21 (65.63)	0.34 (0.18–0.51)	0.28 (0.08, 0.48)	0.45	0.25	2.2/4.0
	Yes	48 (62.34)/ 29 (37.66)	0.62 (0.52, 0.73)				

^*^P < 0.05.

In a multivariable analysis adjusted by sex (OR = 2.56, 95%CI = 1.03–6.37, *P* = 0.04), age (OR = 1.02, 95%CI = 0.99–1.05, *P* = 0.09) and pain duration (OR = 0.67, 95%CI = 0.42–1.08, *P* = 0.10), the disability points score assessed with GCPS at first visit was positively related to acute to chronic transition at 3 months of follow-up (OR = 1.29, 95%CI = 1.06–1.57, *P* = 0.01). This association was not modified by sex (β = 0.14, *P* = 0.49), and it was not related to the number of days with usual activities limitation, but it was positively associated with interference in daily activities, recreational, social, family activities, and ability to work (Table 4).

**Table 4:** Binary logistic regression analyses assessing the association between disability and the acute to chronic transition at 3 months of follow-up.

**Potential risk factor**	**Category**	**Transition/** **no transition**	**Crude model**	***P*-value**	4 Multivariable models*	***P*-value**
			**OR (95% CI)**		**OR (95% CI)**	
Days with activity limitation	Mean (SD)	1.38 (1.43)/ 1.14 (1.38)	1.13 (0.86–1.48)	0.38	1.25 (0.93–1.68)*	0.14
Interference on daily activities	Mean (SD)	4.15 (3.10)/ 2.14 (2.68)	1.27 (1.10–1.46)	0.001	1.33 (1.14–1.56)*	0.003
Interference on recreational, social and family activities	Mean (SD)	4.03 (3.23)/ 2.02 (2.69)	1.25 (1.09–1.43)	0.001	1.33 (1.14–1.56)*	0.004
Interference on ability to work	Mean (SD)	3.61 (3.33)/ 1.82 (2.46)	1.23 (1.07–1.41)	0.003	1.33 (1.13–1.56)*	0.005

^*^Due to multicollinearity (r > 0.84), it was generated 4 multivariable models adjusted by sex (ORs = 2.19, 2.51, 2.82, 2.75, P > 0.08), age (ORs = 1.02, 1.03, P > 0.6), and pain duration (ORs = 0.72, 0.69, 0.68, 0.64, P > 0.07).

When treating the screening score assessed at first-visit as a continuous variable, higher scores were related to the acute to chronic transition (OR_crude_ = 1.32, 95%CI = 1.05–1.66, *P* = 0.02; OR_adjusted_ = 1.37, 95%CI = 1.08–1.76, *P* = 0.01) than those with a lower score. This likelihood was not modified by sex (β = 0.27, *P* = 0.30). However, when treating the screening data as binary, we found that patients with a positive screener (yes = ≥3), did not present an increased transition risk than those with negative score (no ≤ 3) (OR_crude_ = 1.91, 95%CI = 0.76–4.77, *P* = 0.17, OR_adjusted_ = 2.33, 95%CI = 0.86–6.34, *P* =0.10; RR = 1.38, 95%CI = 0.85–1.85, *P* > 0.05).

Table 5 shows that pain that it is always present measured at first-visit was statistically significant related to the transition from acute to chronic pain-related TMD at 3 months of follow-up. We also found that this variable was associated with the transition risk (RR = 1.56, 95%CI = 1.05–1.97, *P* < 0.05). A borderline OR (Table 5) and RR (1.66, 95%CI = 0.97–2.27, *P* > 0.05) were found between pain or stiffness in the jaw on awakening measured at first-visit and the transition at 3 months of follow-up. In addition, based on the adjusted attributed fraction, our results suggest that a large percentage of the transition outcome was attributable to worst and average pain always present, and pain or stiffness of the jaw on awakening, both in the last 30 days (Table 3). Finally, our findings suggest that if 3 patients on average are exposed to pain intensity risk factors and 4 to pain always present and pain or stiffness at first-visit, 1 patient would have the transition from acute to chronic pain at 3 months of follow-up.

**Table 5:** Binary logistic regression analyses assessing the association between TMD-Pain screener items and the acute to chronic transition at 3 months of follow-up.

**Potential risk factor**	**Category**	**Transition/** **no transition**	**Crude model**		Multivariable model*		Multivariable model**	
			**OR (95% CI)**	***P*-value**	**OR (95% CI)**	***P*-value**	**OR (95% CI)**	***P*-value**
Pain always present	Comes and goes	23 (41.07)/ 33 (58.93)	1 (reference)	0.01	1 (reference)	0.04	1 (reference)	0.03
	Is always present	32 (65.31)/ 17 (35.69)	2.70 (1.22–5.97)		2.48 (1.04–5.90)		2.55 (1.08–6.00)	
Pain or stiffness on the jaw on awakening	No	11 (34.38)/21 (65.63)	1 (reference)	0.009	1 (reference)	0.14	1 (reference)	0.06
	Yes	48 (62.34)/ 29 (37.66)	3.16 (1.33–7.49)		2.11 (0.78–5.90)		2.50 (0.96–6.53)	
Chewing	No	12 (50.0)/ 12 (50.0)	1 (reference)	0.64	Not included	
	Yes	47 (55.29)/ 38 (44.71)	1.24 (0.50–3.06)			
Opening	No	12 (42.86)/ 16 (57.14)	1 (reference)	0.17	Not included	
	Yes	47 (58.0)/ 34 (41.98)	1.84 (0.77–4.39)			
Jaw habits	No	20 (48.78)/ 21 (51.22)	1 (reference)	0.39	Not included	
	Yes	39 (57.36)/ 29 (42.65)	1.41 (0.64–3.08)			
Other activity	No	14 (40.0)/ 21 (60.0)	1 (reference)	0.04	1 (reference)	0.21	Not included
	Yes	45 (60.81)/ 29 (39.19)	2.33 (1.02–5.29)		1.84 (0.71–4.80)		

^*^Model adjusted by age (OR = 1.07, P = 0.89), sex (OR = 1.57, P = 0.34) and pain duration (OR = 0.72, P = 0.20).

^**^Model adjusted by age (OR = 1.22, P = 0.67), sex (OR = 1.57, P = 0.33) and pain duration (OR = 0.72, P = 0.19).

**Figure 2:**
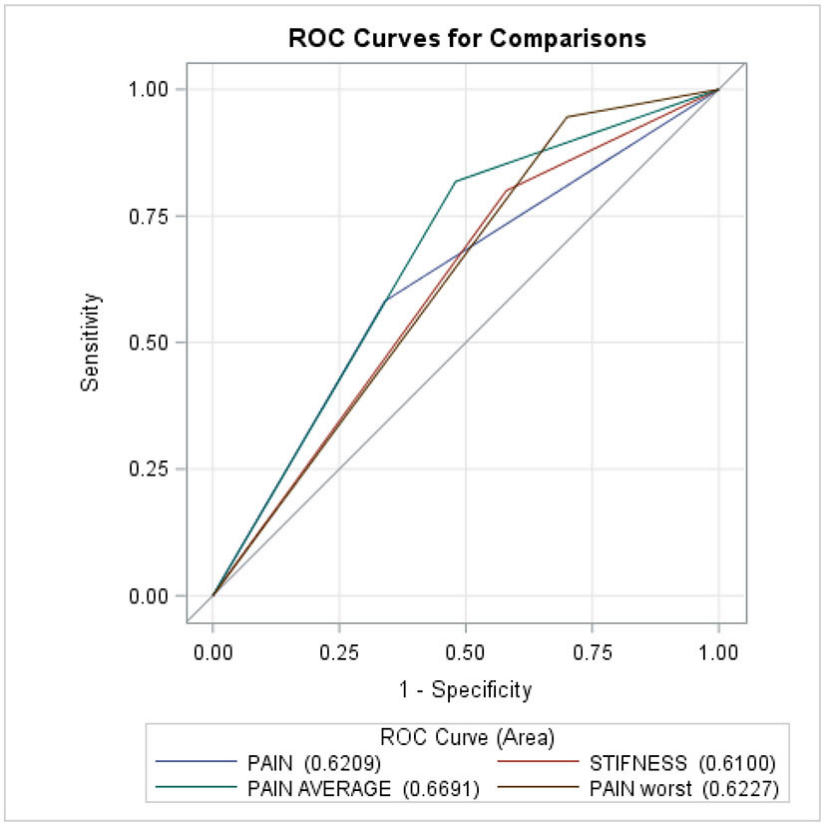
ROC curves for comparisons.

Figure 2 and Table 6 show that the multivariable logistic regression models predicting the transition from acute to chronic pain-related TMD were acceptable, ranging from 61 to 67%.

**Table 6:** ROC model.

	**Mann–Whitney**			
	**Area**	**Standard error**	**95% Wald**	
			**confidence**	**limits**
Pain always present	0.6209	0.0477	0.5275	0.7143
Pain or stiffness on awakening	0.6100	0.0445	0.5227	0.6973
Moderate or severe average pain	0.6691	0.0443	0.5823	0.7559
Moderate or severe worst pain	0.6227	0.0362	0.5518	0.6937

Model adjusted by age, sex and pain duration.

## Discussion

The results of this prospective cohort study are that the transition from acute to chronic pain-related TMD is common, and that CPI, and more specifically moderate to severe average pain, interference with daily, recreational, social or family activities, ability to work, pain always present, in the last 30 days—all assessed at first-visit—were associated with the transition risk from acute to chronic pain at the 3rd month follow-up. Worst pain intensity and pain or stiffness in the jaw on awakening were associated with the transition outcome in the crude analysis. However, when these analyses included the potential risk factors and confounders, significant ORs did not remain. We found a moderately positive correlation between average and worst pain intensities, which in turn contributed to a decrease of the magnitude of the ORs. It should be noted that the multivariable analysis results show that the magnitude of the ORs was >2.4, the 95% CIs were right skewed, and the *P*-values were < 0.09, suggesting that moderate to severe worst pain intensity and pain or stiffness in the jaw on awakening were related to acute to chronic transition at 3 months of follow-up. Moreover, greater attributable fractions—the excess of risk that can be attributed to the exposure—were found with moderate to severe worst pain, average pain, pain always present, and pain or stiffness in the jaw on awakening.

The positive association between pain intensity and transition risk findings (Tables 1, 2) agrees with other studies that also found that pain intensity contributes to acute to chronic pain transition regardless of the definition of acute pain (23–28), or chronic pain (26–28). CPI at first-visit was associated with the transition at 6-month follow-up and with the persistence of pain-related TMD (10, 11, 29, 30). Interferences in daily activities, recreation, social, and family activities contribute to the transition risk (Table 3). These results are parallel with others that found a borderline OR at 6 months of follow-up with GCPS III or IV (13). Unfortunately, no study assessed if specific pain characteristics and oral movements were associated with the transition from acute to chronic pain. Our findings suggest acute pain and interferences impact the central dysregulation mechanisms increasing the transition risk.

Our study has various limitations. First, measurement error in self-reported potential risk factors should be considered. However, as the instruments used are valid and reliable, the likelihood of error should be small. Furthermore, patients with pain at 3 months of follow-up may recall higher CPI, disability, and screening point scores, on their first-visit than those without (recall bias). However, we suggest that reporting errors at first-visit were independent of CPI reporting errors at 3 months of follow-up, since not all potential risk factors (e.g., current pain, worse pain, pain and stiffness, jaw habits) were related to the transition. Second, since 10% of the patients did not complete the 3rd month follow-up there is a possibility, albeit low, of selection bias. Third, the association between the study outcome and potential risk factors was not adjusted by the treatment received by the subject. Fourth, it was not possible to stratify the analyses per enrollment site due to the small sample size. Fifth, the relationship between potential risk factors and outcomes may be biased by unmeasured confounding variables that we are not currently aware of. Sixth, only age and sex were assessed among the demographic variables. Seventh, the sample size of the acute cohort was small.

This is an ongoing cohort study assessing the risk factors for the transition from acute to chronic pain. Prior to the start of the study, a sample size calculation was performed. Because there is no data in the TMD literature for expected outcome and frequencies of the potential risk factors among those with and without an outcome, we performed the estimation using the percentage of outcome (acute to chronic transition) among those exposed and non-exposed of 10 and 40%, respectively. We considered that at least 30% of the difference between these percentages would be clinically significant. Based on these percentages, 62 participants in each group would yield a power of 80% using a two-sided test with an alpha (false positive rate) equal to 0.05. The dropout-adjusted sample size was 36 per group (72 total sample size = 64/1- anticipated dropout rate of 10%). The actual sample sizes of this cohort study for these analyses vary with the specific exposure (Tables 2–5). Power evaluation was based on the *P* > 0.05, confidence interval and magnitude of the OR; if *P* > 0.05 and the confidence interval (which includes “no effect = 1”) does not include clinically meaningful OR (>2), then the study was powered adequately for that outcome. Thus, the results indicated that the study did not have sufficient power for the worst pain intensity and pain or stiffness analyses.

The methodology used has several strengths. First, we estimated all study outcomes prospectively. With this study design, it is almost certain that risk factors or outcome misclassifications are non-differential and would attenuate estimates of association. Second, we assessed whether the ORs would remain when the analyses were adjusted by other risk factors or covariates such as sex. These analyses are essential in view of possible sex differences in pain physiology and clinical outcomes (31–33). Third, we calculated RR based on the adjusted OR, and the adjusted attributable risk factors.

This study has several clinical implications. We found that specific questions from both the self-reported symptoms and TMD screener were related to the transition and thus needed to be considered both in the evaluation and the management of pain-related TMD. Cases exposed to risk factors should receive personalized TMD treatment to prevent the transition.

## Conclusion

In conclusion, the current study demonstrates that CPI, moderate to severe average pain intensity, pain always present, and interferences are related to the acute to chronic transition risk.

## Data availability statement

The raw data supporting the conclusions of this article will be made available by the authors, without undue reservation.

## Ethics statement

The studies involving human participants were reviewed and approved by Institutional Review Board, Faculty of Medicine and Health Sciences, McGill University (IRB # A12-M113-14A). The patients/participants provided their written informed consent to participate in this study.

## Author contributions

AV and MG took part in designing the study. AV, FS, ZK, JB, SE, and MG collected the data. AV conducted the statistical analyses. AV, JB, and SE wrote the manuscript. All authors revised and approved the manuscript.

## Funding

This research received funding from the Quebec Pain Research Network and Le Réseau de Recherche en Santé Buccodentaire et Osseuse.

## Conflict of interest

The authors declare that the research was conducted in the absence of any commercial or financial relationships that could be construed as a potential conflict of interest.

## Publisher's note

All claims expressed in this article are solely those of the authors and do not necessarily represent those of their affiliated organizations, or those of the publisher, the editors and the reviewers. Any product that may be evaluated in this article, or claim that may be made by its manufacturer, is not guaranteed or endorsed by the publisher.

## Footnotes

^1^Available online at: http://grants.nih.gov/grants/guide/rfa-files/RFA-DE-11-001.html.
